# Current Available Computer-Aided Detection Catches Cancer but Requires a Human Operator

**DOI:** 10.7759/cureus.12177

**Published:** 2020-12-19

**Authors:** Florentino Saenz Rios, Giri Movva, Hari Movva, Quan D Nguyen

**Affiliations:** 1 Department of Radiology, University of Texas Medical Branch, Galveston, USA; 2 School of Medicine, University of Texas Rio Grande Valley, Edinburg, USA

**Keywords:** artificial intelligence, machine learning, breast, cancer, mammogram

## Abstract

Introduction: This study intends to show that the current widely used computer-aided detection (CAD) may be helpful, but it is not an adequate replacement for the human input required to interpret mammograms accurately. However, this is not to discredit CAD’s ability but to further encourage the adoption of artificial intelligence-based algorithms into the toolset of radiologists.

Methods: This study will use Hologic (Marlborough, MA, USA) and General Electric (Boston, MA, USA) CAD read images provided by patients found to be Breast Imaging Reporting and Data System (BI-RADS) 6 from 2019 to 2020. In addition, patient information will be pulled from our institution’s emergency medical record to confirm the findings seen in the pathologist report and the radiology read.

Results: Data from a total of 24 female breast cancer patients from January 31st 2019 to April 31st 2020, was gathered from our institution’s emergency medical record with restrictions in patient numbers due to coronavirus disease 2019 (COVID-19). Within our patient population, CAD imaging was shown to be statistically significant in misidentifying breast cancer, while radiologist interpretation still proves to be the most effective tool.

Conclusion: Despite a low sample size due to COVID-19, this study found that CAD did have significant difficulty in differentiating benign vs. malignant lesions. CAD should not be ignored, but it is not specific enough. Although CAD often marks cancer, it also marks several areas that are not cancer. CAD is currently best used as an additional tool for the radiologist.

## Introduction

As society becomes more advanced, so does the technology that accompanies it; as software and the field of artificial intelligence and machine learning begin to evolve, so must society. The most significant point against automation’s progress is the idea that it can replace the complex human role in the process [1,2]. In the field of diagnostic radiology there is a common distrust in Computer-Aided Detection (CAD) with the underlying reasons ranging anywhere from it being a somewhat recent addition to the field in the early 2000s to CAD’s seemingly inability to consistently provide a benefit to the radiologist [3,4]. This study intends to illustrate that current CAD is not an adequate replacement for the human input required to interpret mammograms accurately. This study intends to decrease the current anxiety that both current and future radiologists may have regarding the field’s security. Among women in the United States, breast cancer remains the second most diagnosed type of cancer after skin cancer, with this statistic holding when extrapolated to the worldwide population [5-8]. As such, one cannot neglect the importance of early detection through routine mammography screening, and the early detection of breast cancer is associated with better outcomes [5,6]. However, with each mammogram taken, there must be a radiologist that not only reads it but does so with both high accuracy and at a high volume. CAD-based mammography systems have seen extensive use in the United States following their introduction in 2001; however, since its introduction, CAD has received mixed approval from radiologists regarding its performance [3,4]. One of the more common thoughts is that CAD itself may adversely affect radiologist performance and increase the number of unnecessary biopsies while providing no significant improvement in cancer detection rates [3-9]. However, while CAD has seen noticeable improvements in accuracy, it could be argued that it remains in its infancy 20 years after its introduction. With that in mind, should CAD be partnered with a radiologist to emulate a double read, the overall theoretical cost could remain low [1,10,11]. This would allow an increase in volume to be adopted by the overseeing radiologist [1,10,11]. If efficiently implemented into the healthcare system, this increase in volume could accelerate the decrease in breast cancer mortality by expanding access to high‐quality preventive care at a fraction of the cost [5,6,8,12,13].

## Materials and methods

During the extent of this study, patient information and data on the diagnosis was gathered post patient visit. Inclusion criteria for patient eligibility were that the patient had to be over the age of 18 and had received either craniocaudal (CC) or mediolateral oblique (MLO) mammography during their visit. The patient must have been categorized under Breast Imaging, Reporting & Data System (BI-RADS) as category 6, biopsy-proven malignancy, between January 1st, 2019, and December 31st, 2020. Patient's data was not eligible for analysis in the study if they lacked proper mammogram/breast imaging, if there was an inability to access CAD data for the patient’s imaging, or if they received a pathology report negative for breast cancer (a negative report being classified as BI-RADS category 1 or 2). If the patient met all inclusion criteria and none of the exclusion criteria, their first name, last name, and medical records number (MRN) were recorded within a master data sheet for safekeeping. University of Texas Medical Branch Institutional Review Board issued approval IRB #20-0018.

The number of patients included in the study depended mainly on the volume seen between January 1st, 2019 and December 31st, 2020; however, a sample size between 50 and 100 was predicted based on past trends. Due to the time-limited storage of the CAD data within the institution’s system, only the most recent CAD reads were used throughout this study. Mammogram imaging and CAD reads were obtained and viewed via the Hologic “SecurView® DX” workstation (Hologic, Marlborough, MA, USA) and the included proprietary software. Because of limitations to the CAD data storage, the CAD annotated images of the most recent CAD reads were exported to a password-encrypted disk drive that remained locked within the institution's Department of Radiology. Images were used to record CAD data and were deidentified and disposed of upon completion of the study. Once the CAD reads had been saved, a tally of all CAD markers was recorded onto a master datasheet.

Variables that were recorded onto the master datasheet include patient’s name, MRN, patient identifier code, CAD software used (Hologic or General Electric [Boston, MA, USA]), left and right CC CAD marks, left and right MLO view CAD marks, what was detected in CC CAD, what was detected in MLO CAD, breast density (almost entirely fat, scattered fibroglandular, heterogeneously dense, and extremely dense), radiologic findings of biopsy-proven cancer (mass, calcification, or asymmetry), and the cancer type by pathology report. Each CC and MLO view had their CAD data in terms of the amount of “CAD markings” recorded. The columns “Cancer CC Detected” and “Cancer MLO Detected” were both be used to determine whether or not the left and right breast were found to have one of the five findings, Cancer Hit, Cancer Missed, Cancer Missed w/ Benign Findings, Benign Findings, or No Findings. The two instances of CAD used at the institution included the Hologic and General Electronic’s iteration of the CAD algorithm. The Hologic CAD software markings asterisk, triangle, and cross represent masses, calcification clusters, and a combination mass + calcifications cluster, respectively. Additionally, the General Electric CAD software marked a circle and square representing a mass and calcifications cluster, respectively. Breast density and the final pathology report findings were extracted from the institution’s Epic EMR. Once data gathering has concluded, all variables recorded aside from the patient name and MRN were transferred to a separate data analysis datasheet for statistical analysis. A unique study identifier was used in the data analysis datasheet to help ensure patient privacy and confidentiality.

## Results

Data from 24 female breast cancer patients from January 31st, 2019 to April 31st, 2020, was gathered from the institution's Epic emergency medical record. While the original protocol called for this study to continue for the entire 2020 year, it was decided to end the study prematurely due to the COVID-19 pandemic that began in the spring of 2020.

**Figure 1:**
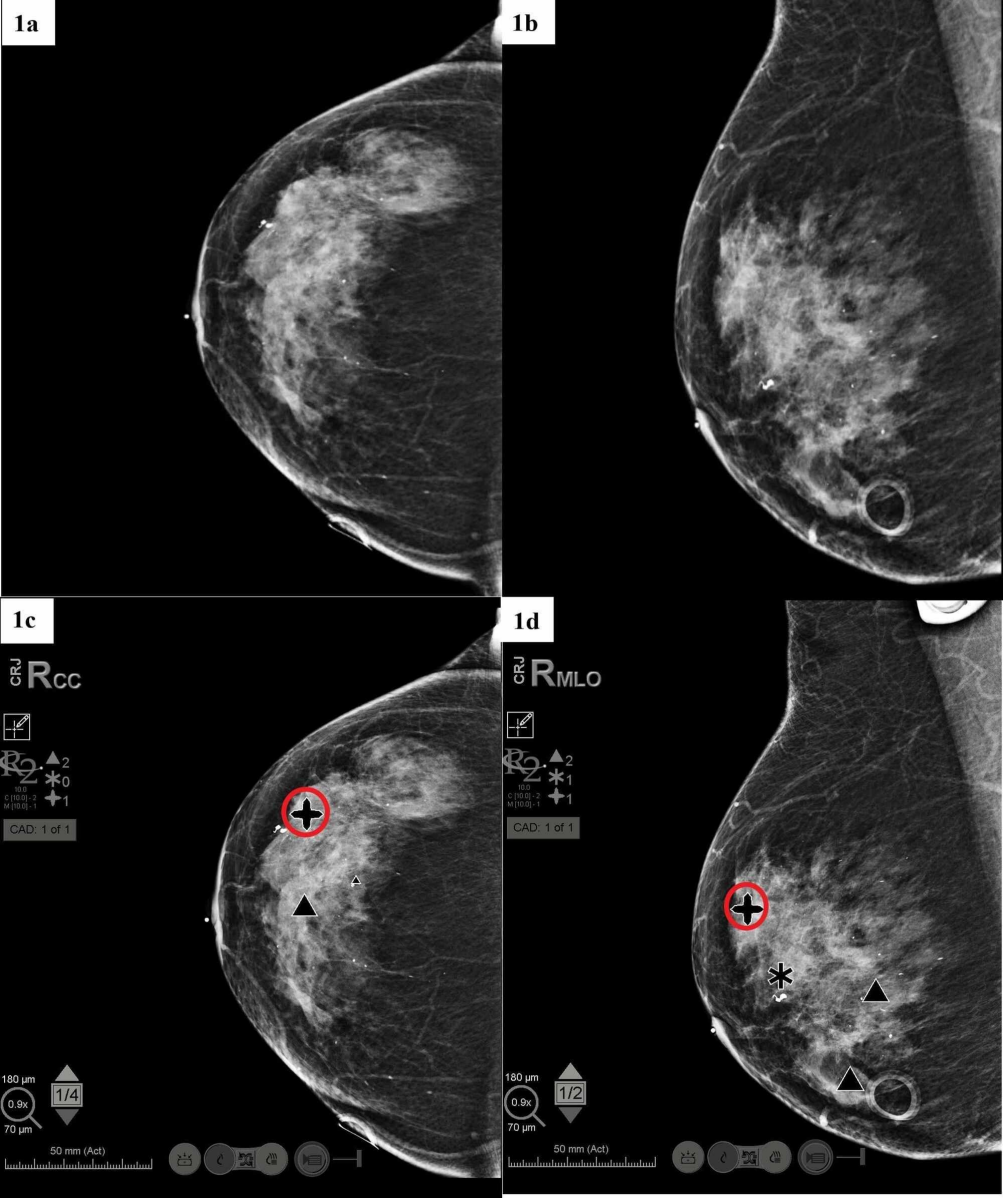
Hologic Computer-aided detection (CAD)* in Dense Breast Patient with Biopsy-Proven Cancer (red circle denotes location of cancer). (a) Right Breast craniocaudal (CC) view without CAD; (b) Right Breast mediolateral oblique (MLO) view without CAD; (c) Right Breast CC view with CAD; (d) Right Breast MLO view with CAD. *Hologic CAD Software Legend: Mass denoted by a black asterisk, calcification clusters denoted by a black triangle, and combinations of mass + calcification clusters are denoted by a black cross.

**Figure 2:**
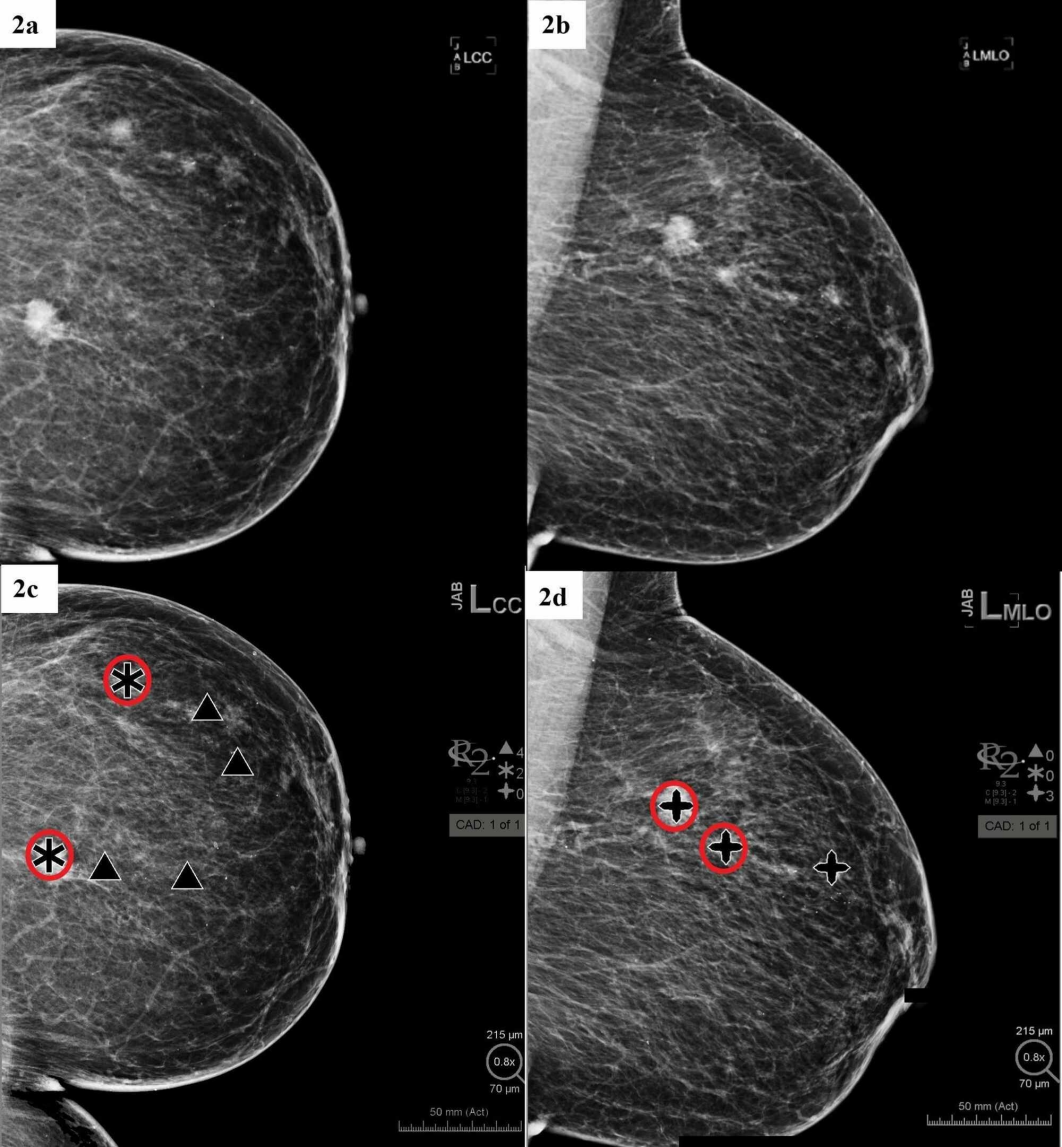
Hologic Computer-Aided Detection (CAD)* in Fatty Breast Patient with Biopsy-Proven Cancer (red circles denote location of cancers) (a) Left Breast craniocaudal (CC) view without CAD; (b) Left Breast mediolateral oblique (MLO) view without CAD; (c) Left Breast CC view with CAD; (d) Left Breast MLO view with CAD. *Hologic CAD Software Legend: Mass denoted by a black asterisk, calcification clusters denoted by a black triangle, and combinations of mass + calcification clusters are denoted by a black cross

Of the 24 patients who met inclusion criteria and underwent CAD imaging, radiologists were able to detect all cancer, and CAD was able to detect most of the cancer. CAD missed the detection of cancer in one of the two views (CC and MLO) for three patients. These data are shown in Table 1, including pathology findings and markings depicted from CAD for each of the patients. The type of breast density also plays a big part in CAD readings. It was found that CAD tends to have a hard time differentiating between malignant or benign in dense breast tissue. In dense breasts (heterogeneously dense or extremely dense), it was found that CAD had an average total marking of 5.33 compared to the fatty breast (fatty and scattered fibroglandular densities) of 4.73, shown in Table 2. An example of dense breasts is shown in Figure 1, and an example of fatty breasts is shown in Figure 2.

**Table 1:** CAD able to detect calcifications and masses, but unable to distinguish the malignant from benign. ^1^Patient has bilateral breast cancer ^2^CAD missed detection in one of the two views (CC or MLO) #1-First biopsy, #2-Second biopsy Computer-aided detection (CAD), Invasive Ductal Carcinoma (IDC), Ductal Carcinoma in Situ (DCIS), Mass (M), Calcifications (C), Asymmetry (A), Mass with Calcifications (MC)

Patient	Radiologist detected	CAD detected	Pathology	Finding	# of CAD calcification markings in breast with cancer	# of CAD mass markings in breast with cancer	Total number of CAD markings
1	Yes	Yes^2^	#1 IDC	M	0	1	1
2	Yes	Yes	#1 IDC, DCIS	M	5	3	8
3	Yes	Yes	#1 IDC, DCIS	M	4	8	12
4	Yes	Yes	#1 DCIS	C	0	3	3
5	Yes	Yes	#1 IDC, DCIS	C	3	3	6
6^1^	Yes	Yes	Left: #1 DCIS Right: #2 DCIS	Left: M Right: M	5	4	9
7	Yes	Yes	#1 IDC, DCIS	C	0	3	3
8	Yes	Yes	#1 IDC	M	0	2	2
9	Yes	Yes	#1 IDC	M	1	3	4
10	Yes	Yes	#1 DCIS	C	1	2	3
11	Yes	Yes	#1 IDC	A	2	3	5
12	Yes	Yes	#1 IDC	M	5	2	7
13	Yes	Yes	#1 IDC #2 IDC	M	3	1	4
14	Yes	Yes	#1 IDC, DCIS	M	0	4	4
15	Yes	Yes	#1 IDC	M	5	2	7
16	Yes	Yes^2^	#1 IDC, DCIS	M	0	1	1
17	Yes	Yes	#1 IDC	MC	2	4	6
18	Yes	Yes	#1 IDC #2 IDC, DCIS	MC	4	5	9
19	Yes	Yes	#1 IDC	M	0	2	2
20	Yes	Yes	#1 IDC	M	0	3	3
21	Yes	Yes	#1 IDC	MC	0	4	4
22	Yes	Yes^2^	#1 IDC #2 IDC	M	1	3	4
23	Yes	Yes	#1 DCIS #2 IDC	M	2	2	4
24^1^	Yes	Yes	Left: #1 IDC, Right: #2 IDC	Left: M Right: MC	5	3	8

**Table 2:** Computer-aided detection (CAD) marks less findings in fatty breasts (fatty and scattered fibroglandular densities) than dense breasts (heterogeneously dense and extremely dense breasts). This means CAD has a harder time and overcalls more in dense breasts. ^1^Patient has bilateral breast cancer

Patient with dense breasts	Number of CAD calcification markings in breast with cancer	Number of CAD mass markings in breast with cancer	Total number of CAD markings
1	0	1	1
2	5	3	8
3	4	8	12
4	0	3	3
5	3	3	6
6^1^	5	4	9
7	0	3	3
8	0	2	2
9	1	3	4
Average number of markings in dense breast	2	3.3	5.3
Patient with fatty breasts	Number of CAD calcification markings in breast with cancer	Number of CAD mass markings in breast with cancer	Total number of CAD markings
10	1	2	3
11	2	3	5
12	5	2	7
13	3	1	4
14	0	4	4
15	5	2	7
16	0	1	1
17	2	4	6
18	4	5	9
19	0	2	2
20	0	3	3
21	0	4	4
22	1	3	4
23	2	2	4
24^1^	5	3	8
Average number of markings in fatty breast	2	2.7	4.7

Analysis of each imaging view (Right CC, Left CC, Right MLO, Left MLO) was conducted to obtain probability values. Probability values were determined from each specific imaging view from the combined interpretation from both radiology and pathology readings. Data were analyzed through the use of Fisher’s exact test. Fisher’s exact test revealed p=0.0417 for right CC CAD interpretation, p=1 for left CC interpretation, p=0.0417 for right MLO CAD interpretation, and p=0.0345 for left CAD interpretation. The probability value for the overall CAD interpretation was conducted using the Chi-square test. It was determined from both the combined interpretation from both radiology and pathology readings for all the views. All values revealed a significant correlation except the left CC CAD interpretation. Despite the small sample size, results showed to be significant in the regard that CAD still needs a human operator to detect cancer.

## Discussion

Artificial intelligence-based algorithms that incorporate machine learning and deep learning are drawing a lot of interest amongst radiologists and the public. However, at this point, those algorithms are not currently widely adopted by breast imaging centers across the nation. The current widely used CAD technology is still too unrefined at the moment. Artificial intelligence-based algorithms improving upon the current CAD would require further research in addition to further investigations into how its implementation in a clinical setting affects patient care. McKinney et al. support this by noting that clinical trials are needed for AI; however, they believe that AI has a role in the future in aiding early detection of breast cancer once refined [1,7,14,15]. McKinney et al.’s [15] study illustrated that CAD is capable of excellent results in controlled testing, but the opposite may occur in real-world scenarios due to various factors including, but not limited to, the radiologists ignoring or misusing CAD due to the high frequency of marks detected that showed no signs of cancer [3,4,7,14] which result in a large number of callbacks [7,14].

Regarding CAD’s effect on radiologist’s reads, Du-Crow et al. [1] showed that observer sensitivity was significantly greater for markers detected by CAD compared to the same markers without CAD (t51=6.56, p<0.001); with no significant difference between no-CAD and CAD conditions on markers that were not detected by CAD. This shows that CAD itself may play a significant role in augmenting the radiologist’s ability as a tool. The data shows that while CAD’s algorithm can detect a potential instance of breast cancer, CAD is not currently able to differentiate between benign and malignant cases of breast cancer. Due to this lack of differentiation, CAD detects many benign findings that untrained eye could be falsely reported as cancer requiring further workup. In working up these cases, unnecessary harm and financial burden may be placed upon the patient through an increase in unnecessary biopsies, and all the additional imaging and procedural cost needed to confirm the findings are non-cancerous [9]. Winch et al. [9] concluded that false positives leading to unnecessary biopsy and further testing could cause patients to worry and develop a fear of cancer until the results are returned and discussed. In addition to this, the legal implication and the liability holder of having a CAD-only system in a hospital is a topic that has not been discussed and would not have a clear-cut answer in the policy.

Overall, due to the relative infancy of CAD, the diversity of the real-world setting, in addition to the program’s inexperience with these demographics, has allowed conflicting information regarding the accuracy of CAD to be published. For example, Lehman et al. [3,4] demonstrate that there was no improvement in the diagnostic accuracy of CAD from a sample of extensive data from the US Breast Cancer Surveillance Consortium registry of clinical mammography interpretations. However, another study on digital breast tomosynthesis CAD system found that detected a good amount of breast cancers that presented via masses or microcalcification clusters (89%, 99 of 111) with an acceptable false-positive rate. While it may be impossible to truly understand whether these conflicting views on CAD are caused by varying patient population or differing technology, further studies are certainly needed to explore the interactions of radiologists and CAD systems [16].­­

## Conclusions

Technology will continue to advance, and artificial intelligence will improve. However, machines replacing humans is unlikely because studies similar to this one will likely continue to show that human plus machine is greater than machine alone. Thus, it is more likely that the technology will add a tool aiding radiologist interpretation rather than replace the radiologist.

Study limitations include factors such as our sample size, which may decrease the significance and generalizability of our results, limitations in the number of CAD data retrieved, differences in detection algorithms between different CAD databases, and the potential decrease of generalizability due to differences in the patient population. Of note, this study had to end prematurely due to the impact of COVID-19 on elective procedures during the Spring, Summer, and Fall of 2020. This limited the number of our patients in the study, which could have provided more support to the significant findings we found with our 24 patients. It may be challenging to be able to compare the various CAD systems used by most institutions accurately; however, it may be possible to alleviate the study limitations of sample size, patient generalizability, and CAD generalizability by creating a multi-institution partnership designed around comparing the CAD systems across the varying institutions. A study on this scale may be difficult to organize due to a lack of logistics and the potential differences the different CAD systems may have in reporting results.
